# Melkersson-Rosenthal Syndrome (MRS): A Case Report

**DOI:** 10.7759/cureus.114327

**Published:** 2026-08-11

**Authors:** Ikram Belatik, Said Kaddouri, Zakaria Chahbi, Hassan Qacif, Mohamed Zyani

**Affiliations:** 1 Internal Medicine, Hôpital Militaire Avicenne, Marrakech, MAR; 2 Infectious Diseases, Hôpital Militaire Avicenne, Marrakesh, MAR

**Keywords:** fissured tongue, granulomatous cheilitis, macrocheilitis, melkersson-rosenthal syndrome, orofacial granulomatosis

## Abstract

Melkersson-Rosenthal syndrome (MRS) is a rare granulomatous disorder classically characterized by recurrent orofacial edema, relapsing peripheral facial palsy, and fissured tongue. However, the complete triad is uncommon, and incomplete forms may create a diagnostic challenge. We report the case of a 48-year-old man with type 2 diabetes mellitus who was admitted with recurrent swelling of both lips and a fissured tongue. The symptoms had begun 15 days earlier with an episode of bilateral macrocheilitis initially attributed to a food allergy. The swelling temporarily improved with antihistamine therapy but recurred one week later. Oral examination revealed marked bilateral lip edema, a fissured tongue, and gingival inflammation, without aphthous lesions, ulceration, xerostomia, or an obvious dental infectious focus. Neurological examination showed no evidence of peripheral facial palsy or facial sensory impairment. Laboratory investigations showed no major abnormalities. Serum calcium and angiotensin-converting enzyme levels were within normal limits. Thoracoabdominopelvic computed tomography and spirometry were unremarkable. QuantiFERON-TB Gold testing and sputum GeneXpert MTB/RIF were negative. A minor salivary gland biopsy was performed to assess granulomatous inflammation and demonstrated chronic non-caseating epithelioid and giant-cell granulomatous sialadenitis without histological evidence of malignancy. Based on the recurrent macrocheilitis, fissured tongue, and compatible granulomatous histology, an incomplete form of MRS was diagnosed. The patient received intralesional triamcinolone acetonide at a dose of 40 mg once monthly for six months, resulting in marked improvement in lip swelling. This case highlights the importance of considering an incomplete form of MRS in patients with recurrent unexplained lip swelling and fissured tongue, even in the absence of facial palsy.

## Introduction

Melkersson-Rosenthal syndrome (MRS) is a rare chronic inflammatory disorder belonging to the spectrum of orofacial granulomatosis. It is classically characterized by the triad of recurrent or persistent orofacial swelling, recurrent peripheral facial nerve palsy, and fissured tongue (lingua plicata) [1,2]. However, the complete triad is observed in only a minority of patients, while monosymptomatic and oligosymptomatic presentations are more frequent and may lead to diagnostic delay [2,3].

Orofacial edema is generally the predominant manifestation and may initially be mistaken for allergic angioedema or infection. The diagnosis is primarily clinical and requires the exclusion of other causes of recurrent orofacial swelling and granulomatous inflammation, particularly Crohn’s disease, sarcoidosis, tuberculosis, dental infection, foreign-body reactions, and angioedema [2,4]. Histopathological examination may reveal non-caseating epithelioid and multinucleated giant-cell granulomas; however, these findings are neither constant nor specific and must be interpreted in conjunction with the clinical presentation [2,4].

We report the case of a 48-year-old man presenting with recurrent bilateral macrocheilitis and a fissured tongue, without facial nerve palsy. Histological examination of a minor salivary gland biopsy revealed non-caseating epithelioid and giant-cell granulomatous sialadenitis. After the main infectious and systemic differential diagnoses were investigated and excluded, an incomplete form of MRS was diagnosed. The patient experienced marked clinical improvement following monthly intralesional triamcinolone acetonide injections.

## Case presentation

A 48-year-old man with a history of type 2 diabetes mellitus treated with oral antidiabetic agents and previous chronic tobacco use was admitted to our internal medicine department with swelling of the lips and oral mucosa, accompanied by oral numbness and a fissured tongue.

The symptoms had begun 15 days before admission with the sudden onset of bilateral macrocheilitis. During an initial visit to the emergency department, the swelling was attributed to a presumed food allergy. The patient was treated with cetirizine (Cetiral®) 10 mg once daily, resulting in temporary clinical improvement. He reported no other medication use or self-medication before admission, apart from his regular metformin treatment for type 2 diabetes mellitus. However, the lip swelling recurred one week later, prompting further evaluation.

The patient reported no fatigue, fever, weight loss, arthralgia, gastrointestinal symptoms, or cutaneous manifestations suggestive of an underlying inflammatory, systemic, or neoplastic disorder. He had no previous history of peripheral facial palsy.

**Figure 1:**
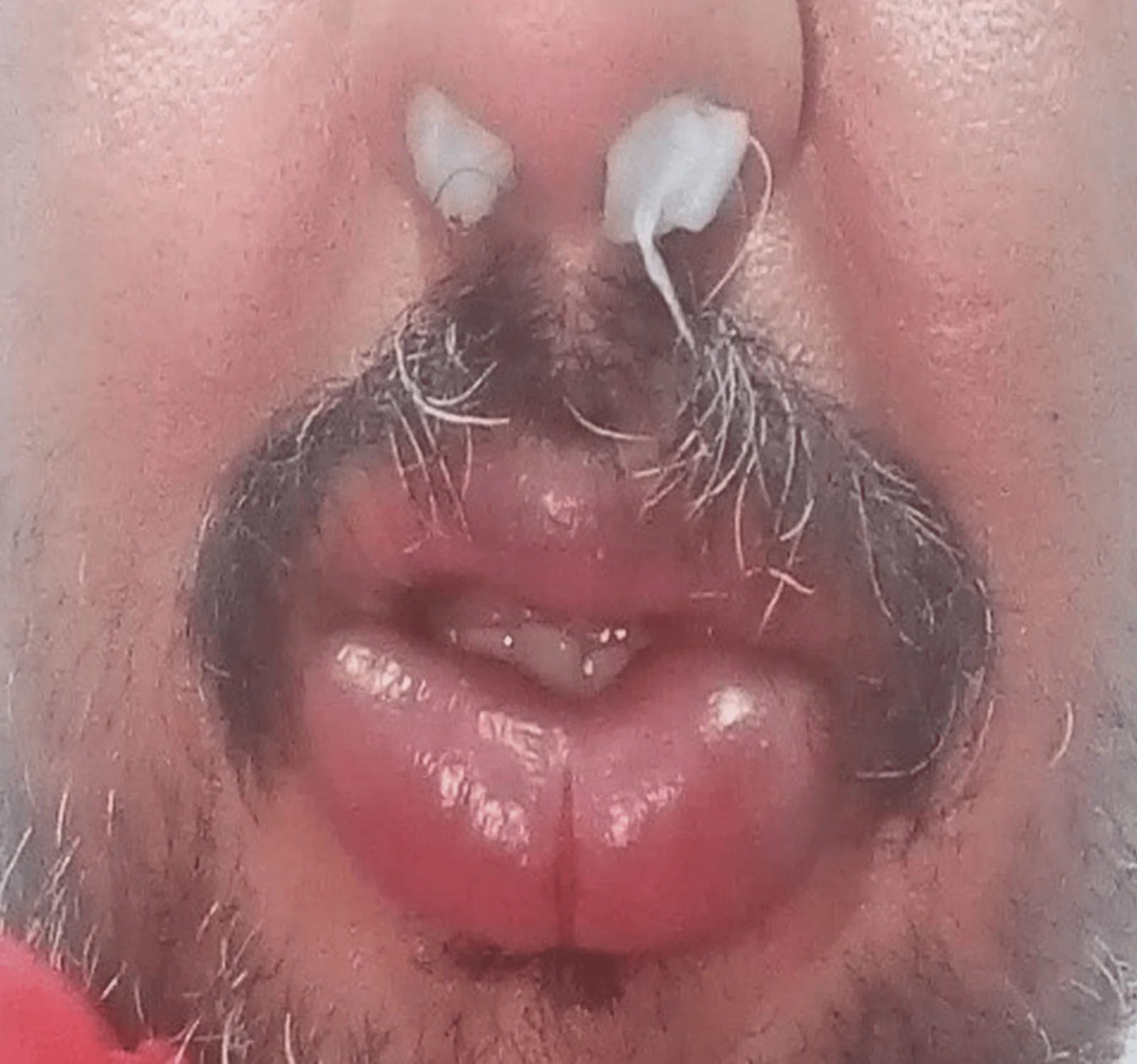
Bilateral lip swelling

**Figure 2:**
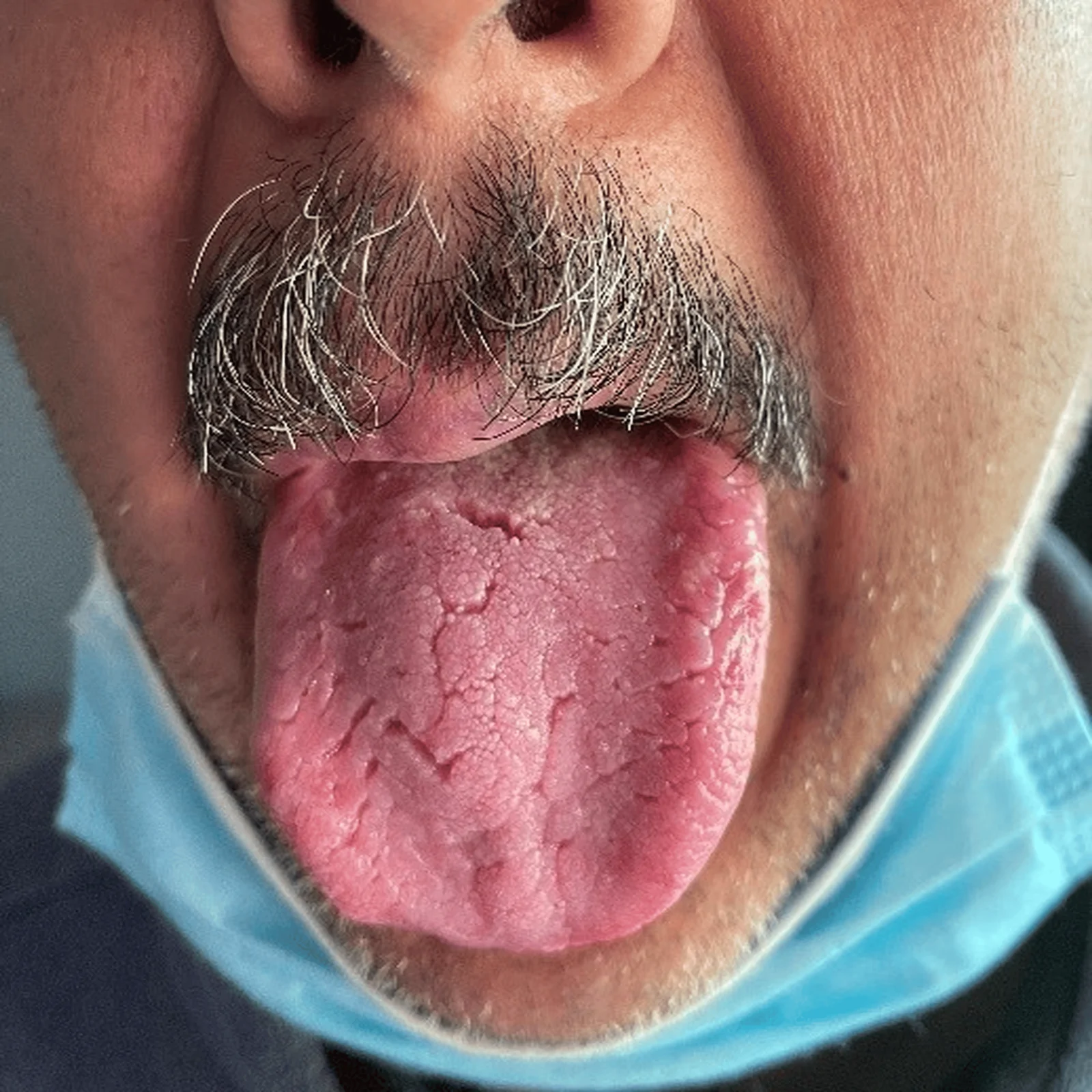
Fissured tongue (lingua plicata)

Oral examination revealed marked edema involving both lips (Figure 1), a fissured tongue (Figure 2), and gingival inflammation. There were no aphthous lesions, mucosal ulcerations, xerostomia, or clinically evident dental infectious focus. Neurological examination showed no evidence of peripheral facial nerve palsy. The Bell phenomenon and Souques’ eyelash sign were absent, and no facial sensory deficit was identified. The remainder of the physical examination was unremarkable.

Laboratory investigations revealed only mild inflammatory changes. The diagnostic work-up was primarily aimed at excluding systemic, infectious, and granulomatous conditions that may mimic MRS. In particular, normal serum calcium and angiotensin-converting enzyme levels provided no laboratory support for sarcoidosis, while negative QuantiFERON-TB Gold and sputum GeneXpert MTB/RIF tests argued against tuberculosis. The remaining hematological and biochemical investigations were unremarkable. The complete laboratory and microbiological results are summarized in Table 1.

**Table 1:** Laboratory and microbiological investigations

Investigation	Result	Unit	Reference range
Hemoglobin	13.2	g/dL	13.0-17.0
Platelet count	220	×10⁹/L	150-400
Lymphocyte count	2.5	×10⁹/L	1.0-4.0
C-reactive protein	10	mg/L	<5
Serum calcium	2.30	mmol/L	2.20-2.60
Angiotensin-converting enzyme	25	U/L	20-80
Urea	3.2	mmol/L	2.5-7.5
Creatinine	80	µmol/L	60-110
Alanine aminotransferase	10	U/L	<40
Aspartate aminotransferase	15	U/L	<40
Alkaline phosphatase	50	U/L	40-130
Urine culture	Sterile	-	No bacterial growth
Sputum GeneXpert MTB/RIF	Negative	-	Negative
QuantiFERON-TB Gold	Negative	-	Negative

As part of the etiological work-up, thoracoabdominopelvic computed tomography was performed to investigate systemic granulomatous disease, including sarcoidosis, and to screen for an underlying neoplastic process; no abnormalities were identified. Spirometry was also performed as part of the assessment for possible pulmonary involvement and was unremarkable.

A minor salivary gland biopsy was performed to investigate the recurrent unexplained orofacial swelling and to assess for histopathological evidence of granulomatous inflammation. Histopathological examination revealed chronic non-caseating granulomatous sialadenitis composed of epithelioid histiocytes and multinucleated giant cells on a lymphocytic inflammatory background. These findings, together with the clinical presentation, supported the diagnosis of MRS.

Based on the recurrent bilateral macrocheilitis, the associated fissured tongue, and the compatible non-caseating granulomatous inflammation, an incomplete form of MRS was diagnosed. The absence of facial nerve palsy explained the incomplete clinical presentation.

The patient was treated with intralesional triamcinolone acetonide, administered at 40 mg to each affected lip once monthly for six months (total dose of 80 mg per treatment session). Given his history of type 2 diabetes mellitus, capillary blood glucose was monitored during hospitalization, and the glycemic profile remained within the target range. Therefore, no adjustment of his metformin therapy was required. The clinical course was characterized by marked improvement in the lip swelling.

## Discussion

MRS is a rare neuro-mucocutaneous granulomatous disorder characterized by considerable clinical heterogeneity. Although it is traditionally defined by the triad of recurrent orofacial edema, recurrent peripheral facial palsy, and fissured tongue, complete forms are uncommon. In a retrospective series of 36 patients, the complete triad was present in only 25%, whereas orofacial involvement was the dominant manifestation [3]. Our patient presented with recurrent bilateral macrocheilitis and a fissured tongue, without current or prior facial palsy, supporting the diagnosis of an incomplete or oligosymptomatic form of MRS.

Orofacial swelling is frequently the earliest and most prominent manifestation. It may initially be intermittent and partially regress spontaneously or after nonspecific treatment, whereas repeated episodes may eventually result in persistent, firm swelling [1,2]. In our patient, the first episode was attributed to a presumed food allergy and temporarily improved following antihistamine therapy. However, recurrence one week later, together with the presence of a fissured tongue, prompted further evaluation. A transient improvement with antihistamines does not establish an allergic mechanism, particularly because MRS may follow a fluctuating course.

Orofacial granulomatosis describes a spectrum of disorders characterized by recurrent or persistent swelling of the lips, face, or oral mucosa associated with non-caseating granulomatous inflammation. This spectrum includes MRS and granulomatous cheilitis, while clinically similar orofacial lesions may also occur in Crohn’s disease, sarcoidosis, dental infections, and other granulomatous disorders [2,4,5]. Granulomatous cheilitis is generally considered a monosymptomatic presentation. In contrast, the association of recurrent macrocheilitis with a fissured tongue, as observed in our patient, is more consistent with an oligosymptomatic form of MRS [2,5].

The diagnosis of MRS remains primarily clinical because no laboratory or histopathological finding is pathognomonic. Histological examination may demonstrate non-caseating epithelioid granulomas, multinucleated giant cells, lymphocytic infiltration, and tissue edema. Nevertheless, granulomas are not invariably present, and their absence does not exclude the diagnosis [2,5]. Conversely, their presence is not specific and requires consideration of other infectious and systemic granulomatous diseases. In our patient, minor salivary gland biopsy demonstrated chronic non-caseating epithelioid and giant-cell granulomatous sialadenitis. This finding provided supportive evidence but was interpreted together with the recurrent macrocheilitis, fissured tongue, and clinical and laboratory/imaging assessment.

The differential diagnosis of recurrent macrocheilitis with granulomatous inflammation includes Crohn’s disease, sarcoidosis, tuberculosis, foreign-body granulomas, dental infection, allergic or hereditary angioedema, and neoplastic disease [4,5]. Granulomatous cheilitis may also be associated with hypersensitivity to certain foods or food additives, including benzoates and cinnamon-containing products, as well as sensitivity to dental restorative materials [4,5]. When no underlying etiology is identified, it may be considered idiopathic [5]. In the present case, thoracoabdominopelvic computed tomography, spirometry, serum calcium, and angiotensin-converting enzyme levels were unremarkable, making clinically active sarcoidosis less likely. However, a normal serum angiotensin-converting enzyme level alone does not exclude sarcoidosis. Tuberculosis was considered less likely because thoracic computed tomography was unremarkable, sputum GeneXpert MTB/RIF and QuantiFERON-TB Gold testing were negative, and histological examination showed no caseous necrosis.

The patient reported no gastrointestinal symptoms suggestive of Crohn’s disease. Nevertheless, the absence of digestive manifestations does not formally exclude subclinical intestinal involvement, because orofacial granulomatosis may occasionally precede the development of gastrointestinal Crohn’s disease [4,6]. Clinical follow-up and further gastrointestinal investigations should therefore be considered if digestive symptoms, weight loss, iron deficiency, or biological inflammation subsequently occur.

The pathogenesis of MRS remains uncertain. Proposed mechanisms include immune dysregulation, genetic susceptibility, delayed hypersensitivity reactions, and infections, but none have been conclusively established [1,4]. Consequently, there is no universally accepted therapeutic protocol. Available evidence is mainly derived from retrospective series, small case series, and case reports, with no robust randomized controlled trials supporting a single treatment strategy [1,7].

Corticosteroids are the most commonly reported treatment, particularly for orofacial edema and facial nerve involvement. Intralesional triamcinolone acetonide is frequently used for localized lip swelling because it permits direct delivery to the affected tissue while limiting systemic exposure [1,5]. Other therapeutic approaches reported in refractory disease include systemic corticosteroids, antibiotics with anti-inflammatory properties, clofazimine, hydroxychloroquine, methotrexate, thalidomide, biological agents, and surgical reduction cheiloplasty. However, therapeutic responses are variable, and the supporting evidence remains limited [1,7,8].

Our patient received intralesional triamcinolone acetonide at a dose of 40 mg into each affected lip once monthly for six months, resulting in marked improvement in lip swelling. Intralesional triamcinolone has been reported as an effective treatment for localized granulomatous cheilitis and orofacial edema. However, the dosing regimens described in the literature vary, and no standardized regimen has been established [1,5]. In our patient, the treatment regimen was adapted to the bilateral and recurrent nature of the lip involvement. Nevertheless, because spontaneous fluctuation and recurrence are characteristic of the disease, prolonged follow-up is required before sustained remission can be established.

The principal limitations of this case are the absence of a reported long-term follow-up period after completion of treatment and the unavailability of the original histopathology slides and photomicrographs because of the time elapsed since the case occurred. Despite extensive efforts, these materials could not be retrieved. Nevertheless, the histopathological findings were documented in the patient’s medical record and, together with the compatible clinical presentation, supported the diagnosis. Furthermore, although the available evaluation did not reveal an infectious, systemic, or neoplastic cause, the absence of gastrointestinal symptoms cannot completely exclude subclinical Crohn’s disease. Continued clinical surveillance is therefore appropriate.

This case emphasizes that MRS should be considered in patients presenting with recurrent unexplained macrocheilitis, especially when associated with a fissured tongue, even in the absence of facial palsy. Recognition of incomplete forms may prevent repeated misdiagnosis as an allergy and facilitate appropriate clinicopathological assessment.

## Conclusions

MRS should be considered in patients presenting with recurrent or persistent lip swelling, particularly when macrocheilitis is associated with a fissured tongue. The absence of facial palsy does not exclude the diagnosis, as incomplete forms are more frequent than the classical triad. Diagnosis relies on integrating clinical findings and compatible histopathological features while excluding infectious, inflammatory, systemic, and neoplastic causes of orofacial granulomatosis. In our patient, monthly intralesional triamcinolone acetonide injections were associated with marked clinical improvement. However, long-term follow-up remains necessary because relapses may occur and systemic manifestations, particularly gastrointestinal or neurological involvement, may develop subsequently.
